# Pharmacist-led counselling intervention to improve antiretroviral drug adherence in Pakistan: a randomized controlled trial

**DOI:** 10.1186/s12879-020-05571-w

**Published:** 2020-11-23

**Authors:** Zeenat Fatima Chatha, Usman Rashid, Sharon Olsen, Fakhar ud Din, Amjad Khan, Komal Nawaz, Siew Hua Gan, Gul Majid Khan

**Affiliations:** 1 Department of Pharmacy, Quaid-i-Azam University, Islamabad, Pakistan; 2 Health and Rehabilitation Research Institute, Auckland University of Technology (AUT), Auckland, New Zealand; 3 School of Pharmacy, Monash University Malaysia, 47500 Bandar Sunway, Selangor Darul Ehsan Malaysia

**Keywords:** ART adherence, HIV, Counselling, Pharmacist intervention, Prevention

## Abstract

**Background:**

Pakistan is facing a growing population of people living with human immunodeficiency (HIV). In this randomized controlled trial, we investigate if a pharmacist-led intervention can increase adherence to antiretroviral therapy (ART) for people living with HIV (PLWH).

**Methods:**

Adults with HIV, who have been taking ART for more than 3 months were randomly assigned to receive either a pharmacist-led intervention or their usual care. Measures of adherence were collected at 1) baseline 2) just prior to delivery of intervention and 3) 8 weeks later. The primary outcomes were CD4 cell count and self-reported adherence measured with the AIDS Clinical Trial Group (ACTG) questionnaire.

**Results:**

Post-intervention, the intervention group showed a statistically significant increase in CD4 cell counts as compared to the usual care group (*p* = 0.0054). In addition, adherence improved in the intervention group, with participants being 5.96 times more likely to report having not missed their medication for longer periods of time (*p* = 0.0086) while participants in the intervention group were 7.74 times more likely to report missing their ART less frequently (*p* < 0.0001).

**Conclusions:**

The findings support the improvement in ART adherence and HIV management.

**Trial registration:**

The trial is registered with Australian New Zealand Clinical Trials Registry (ACTRN12618001882213). Registered 20 November 2018.

**Supplementary Information:**

The online version contains supplementary material available at 10.1186/s12879-020-05571-w.

## Background

The human immunodeficiency virus (HIV) is a global pandemic affecting mostly both low- and middle-income countries [1]. Pakistan, a lower to middle-income country, has seen a rapid growth in HIV epidemic [2]. Some factors contributing to this phenomena include low level of education on HIV transmission, poor infection control practices and insufficient HIV prevention programs, particularly in relation to condom use among sex-workers, male homosexuals, transgenders and intravenous drug-users as well as poor safety on injection practices [2–4]. It is estimated that Pakistan has an estimated 160,000 people living with HIV (PLWH) [5] and the number is growing rapidly, making treatment programs a priority. Recently, some studies from Pakistan reported a low adherence to ART among intravenous and non-intravenous drug users [6]. Similarly, a systematic review was reported earlier to determine the relationship between socioeconomic status and adherence among the HIV patients in low- and middle-income countries demonstrating that a convincing evidence was not established between the two comparative groups [7]. However, another study reported the fact that various factors can influence adherence to ART. These include the disease status, therapy, and affiliation of the patient with the healthcare provider [8].

Fortunately, with the introduction of ART, the status of HIV/AIDS has changed from a lethal to chronic disease [9]. It is hypothesized that in order to achieve HIV viral suppression, adherence rates of at least 82–95% are ideal [10, 11] and should therefore be targeted. However, individuals in low- and middle-income countries are said to be 1.6 times more likely to have suboptimal adherence to ART [12]. In Pakistan specifically, data has indicated that 17% of people taking ART have suboptimal adherence [13] and the majority (81%) of intravenous drug-users miss more than three ART treatments per month [6]. The suboptimal adherence is detrimental since it 1) can increase the risk of progression from HIV to AIDS [14], 2) increase the risk of drug resistance [15] and 3) threaten further spread of the disease [16]. Thus, interventions which can increase adherence to ART are urgently required. Moreover, the WHO drug resistance report 2012 stated that with the increased number of people accessing ART, the prevalence of pre-treatment drug resistance has increased in several low-income and middle-income countries [17]. Available data shows that pre-treatment drug resistance is significantly more prevalent in people with previous ART exposure and leads to virological failure [18].

Counselling is a key strategy that can improve ART adherence by enhancing awareness, emotional support and improving self-efficacy [19]. Pharmacists and other health professionals have similar consultation approaches which tend to vary during follow up. Many studies have suggested that by incorporating pharmacist in the multidisciplinary care, adherence to ART can be improved and virological suppression achieved [20]. Additionally, pharmacist-led interventions also incur some cost savings in terms of ART adherence treatment [21]. In fact, there is no disadvantages of pharmacist-led intervention documented to date. Individualized counselling with a strong educational component can address the cognitive, emotional, social and behavioral factors that impede adherence to ART [22]. Therefore, it is recommended that education is delivered at the beginning of treatment, at a single point during treatment, or on an ongoing basis [23].

Pharmacists who are routinely involved in providing education about medications, have seen a growth in their roles in relation to ART. The roles include medication monitoring, management of drug-related adverse events and adherence counselling [24–29] Indeed, pharmacists 1) are uniquely-placed to provide patients with individualized education and 2) have vital roles in improving ART adherence [28, 30]. People living with HIV who receives pharmaceutical education believe that it is beneficial, particularly for managing drug interactions, side effects and missed doses [31]. The notion is supported by existing systematic review of randomized and non-randomized controlled trials, pre-post comparisons and cohort studies, which indeed confirmed a positive association between pharmacist-led interventions and ART adherence [32]. It is plausible that the pharmacist-led intervention can break bad medication-taking behaviors and underline the importance and benefits of treatments on the patients.

The aforementioned pharmacist-led adherence systematic review research [32] was conducted in high-income countries which may not be applicable to the local setting in Pakistan. In fact, pharmacy-based research in Pakistan remains in its infancy and faces a number of challenges, including developing a clearly-defined role for pharmacists and earning acceptance from other clinicians [33]. Thus, there is a need to expand the role of pharmacy practice in Pakistan, particularly in the area of ART. In this study, we evaluated the effect of a pharmacist-led education and counselling intervention, compared with usual care, on ART adherence and HIV control among individuals with HIV living in Pakistan.

## Methods

### Study design and participants

This is a single-blinded randomized controlled trial (RCT) undertaken between September 2018 and February 2019 at the HIV Care Center, Pakistan Institute of Medical Sciences (PIMS), Islamabad, Pakistan. The inclusion criteria were: HIV positive sero-status, > 18 years of age, taking ART for > 3 months and not enrolled in another treatment adherence program. The exclusion criteria were: having incomplete baseline blood test, pregnancy, or a cognitive impairment that may prevent engagement with the intervention.

### Randomization and masking

Following informed consents and collection of baseline measurements, participants were randomly assigned to either the ‘pharmacist-led intervention’ or ‘usual care’ group, based on a computer-generated block randomization schedule [34]. The randomization codes were concealed in unmarked, sealed envelopes and were allocated by a nurse who was not involved in either the outcome measurement or the intervention delivery. Participants remained blinded to the group and were unaware of the timeframes during which the intervention and usual care groups would receive the pharmacist-led intervention. Overall, the intervention group received the intervention at the start of the study period, whilst the usual care group received the intervention following the conclusion of the study. Cluster of differentiation 4 (CD4) count was collected by assessors who were blinded to the grouping, but measures of adherence were collected by an assessor who is not blinded to the study. Data analysis was completed by a researcher who was blinded to the allocation of the group.

### Procedures

Prior to study enrolment, the participants received a single education and counselling session when their ART was started, led by a physician and administered according to the national guidelines [35]. Then whenever required, especially if ART was changed, a follow-up counselling was provided, this time by a nurse. During the eight-week study period, both groups continued to have access to this nurse-led education if required and collected their medicines from a pharmacy technician.

In addition to the usual care, the intervention group also received the pharmacist-led intervention involving a single face-to-face counselling (30-min session) and education. The intervention was delivered after the collection of baseline measures. The intervention was tailored to each participants’ social and cultural background, their beliefs about the effectiveness of ART and their baseline level of adherence. Intervention fidelity was monitored by a lead clinician at the HIV center, who supervised random intervention sessions on a daily basis. The education was focused on HIV transmission, disease state, cure, treatment resistance, viral load and safe sex. The counselling component focused on personal barriers to taking medication and is aimed at helping participants understand their medication-taking behaviors while acknowledging the actions needed to maintain a high level of adherence. The counselling session included advice on the potential negative impact of diet and supplementary herbs or medicines on the effectiveness of ART.

### Outcomes

The outcome measures were collected at baseline and at the end of the eight-week intervention period. The first primary outcome was CD4 cell count, which provides a measure of HIV disease progression as well as patients’ immune status [36]. The information was collected from the medical records, both on the day of enrolment and at the end of the eight-week study period. Follow up measures were integrated with the participants’ routine visits to the clinics in order to achieve the eight-week period between baseline and follow up.

The second primary outcome was a self-reported adherence to ART, as assessed using section D of the Adult AIDS Clinical Trials Group (AACTG) adherence instrument [37] which has been validated in a resource-limited settings [38]. This section of the AACTG asks the participants on the details of the last time they missed they medication (if any) to be rated within the past week, 1–2 weeks, 2–4 weeks, 1–3 months, > 3 months, or never. Two secondary outcomes were recorded 1) the reasons for having missed the medications (section C, AACTG) and 2) adherence self-efficacy as well as medication beliefs (section A, AACTG). Section C determines how often in the past month, they have non-adherence being rated from ‘often’ to ‘never’ on a four-point Likert scale as well as the possible reasons for non-adherence. Section A asks three questions related to 1) the ability to take medications as directed, 2) beliefs about medication efficacy and 3) beliefs about whether skipped medications may result in ART resistance; rated from ‘not at all sure’ to ‘extremely sure’ on a four-point Likert scale. Participants also completed section B of the AACTG at baseline, to gauge the social support received.

### Sample size

The sample size was calculated in order to detect a statistically significant difference in the mean self-reported adherence (Cohen’d = 0.5) across two time-points within the intervention group using a repeated measures t-test at 80% power and a 5% type-I error-rate. The required sample size was 34 for each group (total 68); however this was elevated to 100 to account for the high dropout-rate in HIV adherence studies [39].

### Statistical analysis

Data were analyzed using R (The R Foundation for Statistical Computing, Vienna,Austria) version 3.5.1 [40]. *MASS* version 7.3–51.1 [41], *stats* version 3.5.1 [40] and *ggplot2* version 3.2.0 [42] were used to build statistical models, perform hypothesis tests and plot data. A detailed statistical analysis report with data visualizations and code snippets for the models is available as a supplementary file. The statistical analysis evaluated the treatment effect (the difference in the outcomes between the intervention and the usual care groups) on the two primary outcomes. The analysis was based on complete cases. However, multiple imputations were not performed as the outcome measure categories were the only cells which had missing data [43]. The data were fitted with linear regression models. A longitudinal approach was adopted by adjusting for the baseline characteristics to cater for the missing data [44]. CD4 cell count data was analyzed using a linear regression model which estimated the difference across the two groups. Self-reported adherence, the reasons for having missed medication, adherence self-efficacy and medication beliefs, were analyzed using three separate models. Each model estimated the effect of the intervention using ordinal logistic regression on the relevant Likert scale.

Treatment effects were estimated as odd ratios, which provide the ratio of the odds of the participants giving a higher response on the relevant Likert scale in the intervention group compared to the usual care group. An odds ratio of 1 suggests that the participants in both groups are equally likely to give a higher response on the scale. All statistical models were adjusted for the baseline outcomes and for covariates including social support (section B of AACTG baseline instrument), age, gender, education, employment, marital status and the cause for HIV. The estimated treatment effects are reported together with their 95% confidence intervals (*p* < 0.05).

## Results

**Fig. 1:**
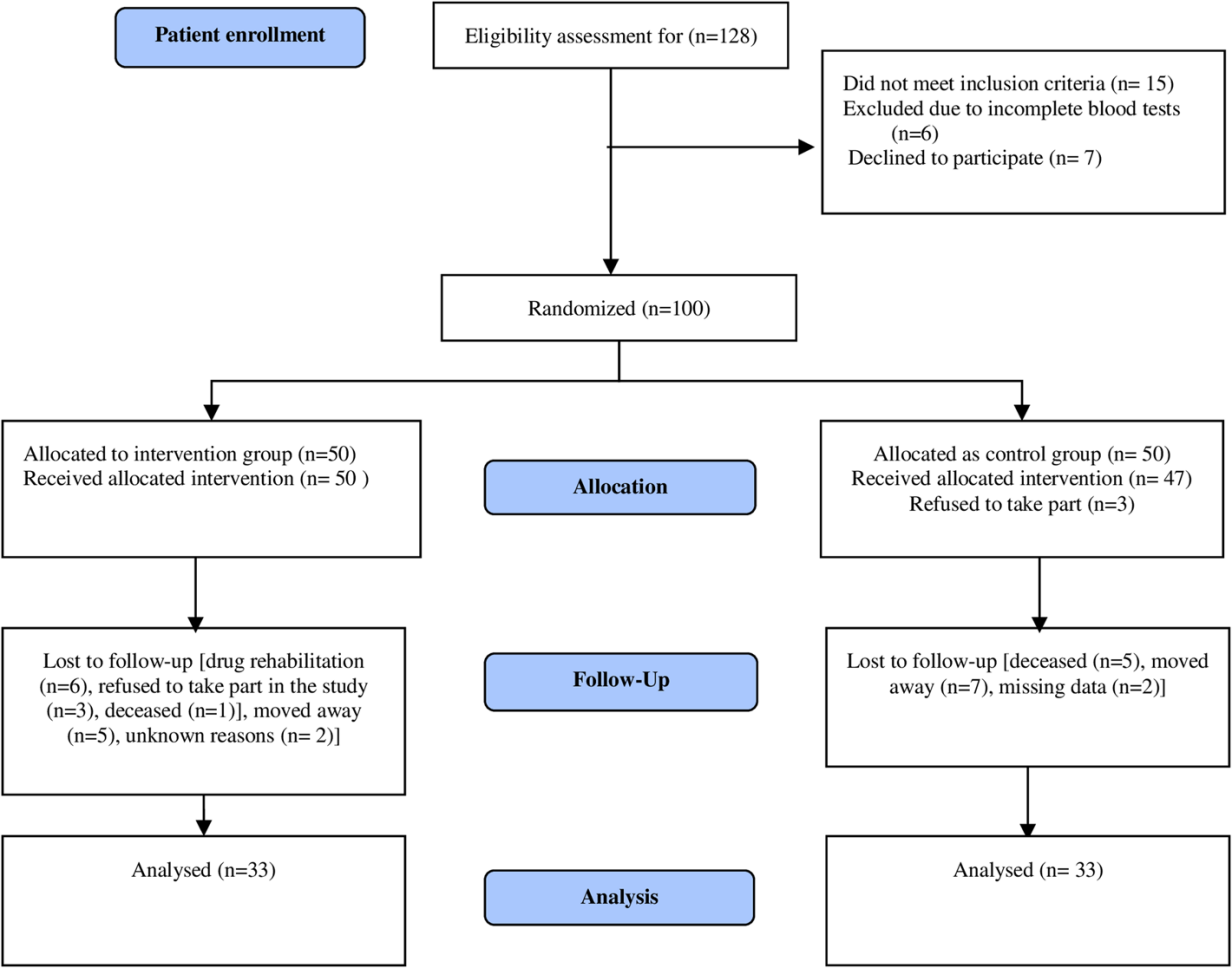
CONSORT diagram. Provides details of enrolment, randomization, group allocation, follow-up and analysis

Figure 1 shows the consolidated standards of reporting trials (CONSORT) diagram from the screening step to study completion. Out of the 128 people screened for participation, 100 were eligible and were randomly assigned to either the intervention group (*n* = 50) or the usual care group (n = 50). All participants in the intervention group received the pharmacist-led intervention, but 17 dropped out or were lost to follow up. In the usual care group, three participants refused to take part immediately after randomization and another 14 dropped out during the intervention period or were lost to follow up. Finally, there were a total of 33 participants in each group.

### Socio-demographic characteristics and social support

The baseline socio-demographic characteristics and self-reported level of support (section B, AACTG) are presented in Tables 1 and 2 respectively. Participants were predominantly male, uneducated and unemployed. The main modes of HIV transmission were sexual transmission and shared needles during drug use. Participants reported moderate levels of social support.

**Table 1:** Baseline sociodemographic characteristics

Sociodemographic characteristics	Usual care	Intervention
Age mean (SD)	31.39 (9.53)	36.18 (12.24)
Gender, n (%)		
Male	18 (54.55)	23 (69.70)
Female	10 (30.30)	10 (30.30)
Transgender	5 (15.15)	NA
Education, n (%)		
Educated	6 (18.18)	6 (18.18)
Uneducated	27 (81.82)	27 (81.82)
Employment, n (%)		
Employed	15 (45.45)	10 (30.30)
Unemployed	18 (54.55)	23 (69.70)
Marital status, n (%)		
Married	18 (54.55)	21 (63.64)
Unmarried	14 (42.42)	11 (33.33)
Divorced	1 (3.0)	1 (3.03)
HIV transmission cause, n (%)		
Sex	23 (69.70)	25 (75.76)
Shared needles	5 (15.15)	5 (15.15)
Other	5 (15.15)	3 (9.09)

**Table 2:** Self-reported level of support

Social support	Usual care N (%)	Intervention N (%)
B1: In general, how satisfied are you with the overall support you get from your friends and family members?		
Very dissatisfied	7 (21.21)	5 (15.15)
Somewhat dissatisfied	10 (30.30)	8 (24.24)
Somewhat satisfied	13 (39.39)	14 (42.42)
Very satisfied	3 (9.09)	6 (18.18)
B2: To what extent do your friends or family members help you remember to take your medication?		
Very dissatisfied	9 (27.27)	4 (12.12)
Somewhat dissatisfied	8 (24.24)	9 (27.27)
Somewhat satisfied	7 (21.21)	10 (30.3)
Very satisfied	9 (27.27)	10 (30.3)

### HIV viral load: CD4 cell count

**Fig. 2:**
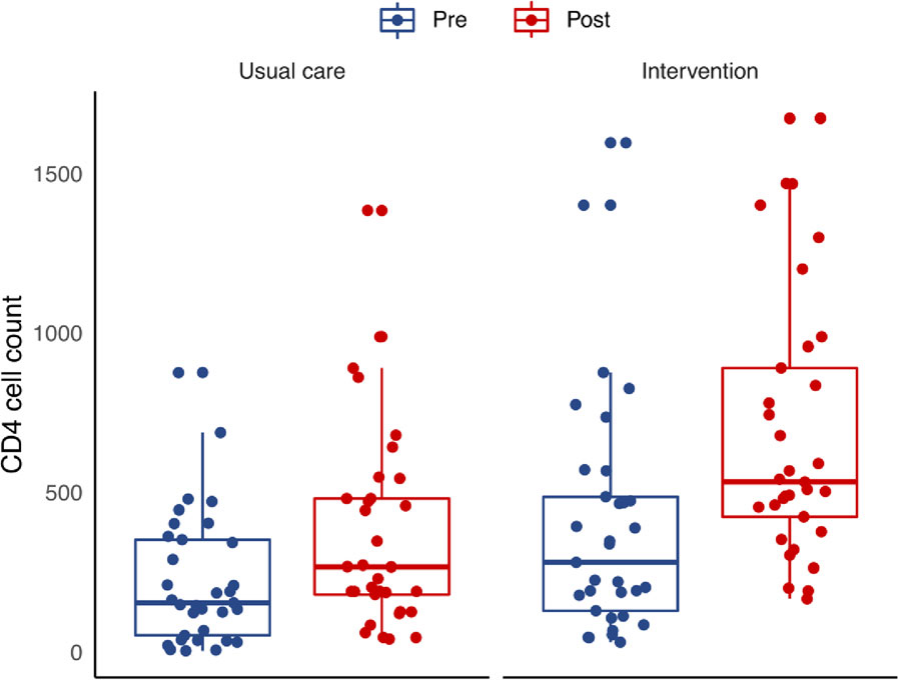
CD4 cell count results. Pre- and post-intervention plots illustrating CD4 cell counts of individual participants (dots) and usual care and intervention groups (boxes with medians and interquartile ranges)

The CD4 cell counts are presented in Fig. 2. The statistical model indicated a statistically significant increase in CD4 cell counts in the intervention group, post-intervention (mean difference = 199.67 [95% CI 61.81–337.53], t [48] =2.91, *p* = 0.0054).

### Self-reported adherence: the last time medication was missed (section D, AACTG)

**Fig. 3:**
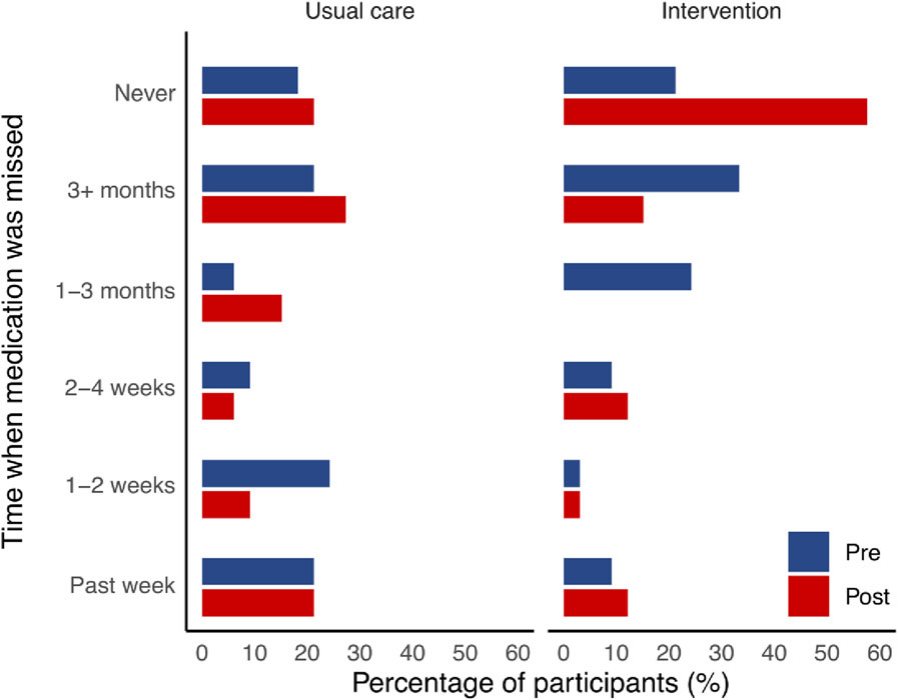
Self-reported adherence results. Pre- and post-intervention plots illustrating self-reported timeframes in which medication was last missed for the usual care and intervention groups

The last time participants missed taking any of their medication(s) is presented in Fig. 3. At post-intervention, there was a 36% increase in the number of participants in the intervention group who ‘never’ missed their medication, compared to only 3% in the usual care group. Additionally, compared to the usual care group, participants in the intervention group were 5.96 times more likely to report a higher response on the time scale which comprised the past week, 1–2 weeks, 2–4 weeks, 1–3 months, 3+ months, or never missed medication (OR [95% CI] = 5.96 [1.57–22.55], z = 2.63, *p* = 0.0086). Moreover, there was a statistically-significant association between higher responses (longer periods of adherence) and social support (B2) (*χ*^2^ [3] =8.44, *p* = 0.0377), such that people with lower levels of social support were more likely to be adherent post-intervention (somewhat satisfied/very dissatisfied OR [95% CI] =0.06 [0.003–1.06]). There was also an association between the cause of HIV transmission and higher responses (*χ*^2^ [2] =9.15, *p* = 0.0103), such that participants who reported sexual transmission as the cause of getting the disease were less likely to adhere to medication compared to those who reported other causes of transmission (other/sex OR [95% CI] =23.14 [2.29–234.12]). For further details regarding effect sizes please refer to the supplementary file.

### Reasons for having missed medication in the past month (section C, AACTG)

**Fig. 4:**
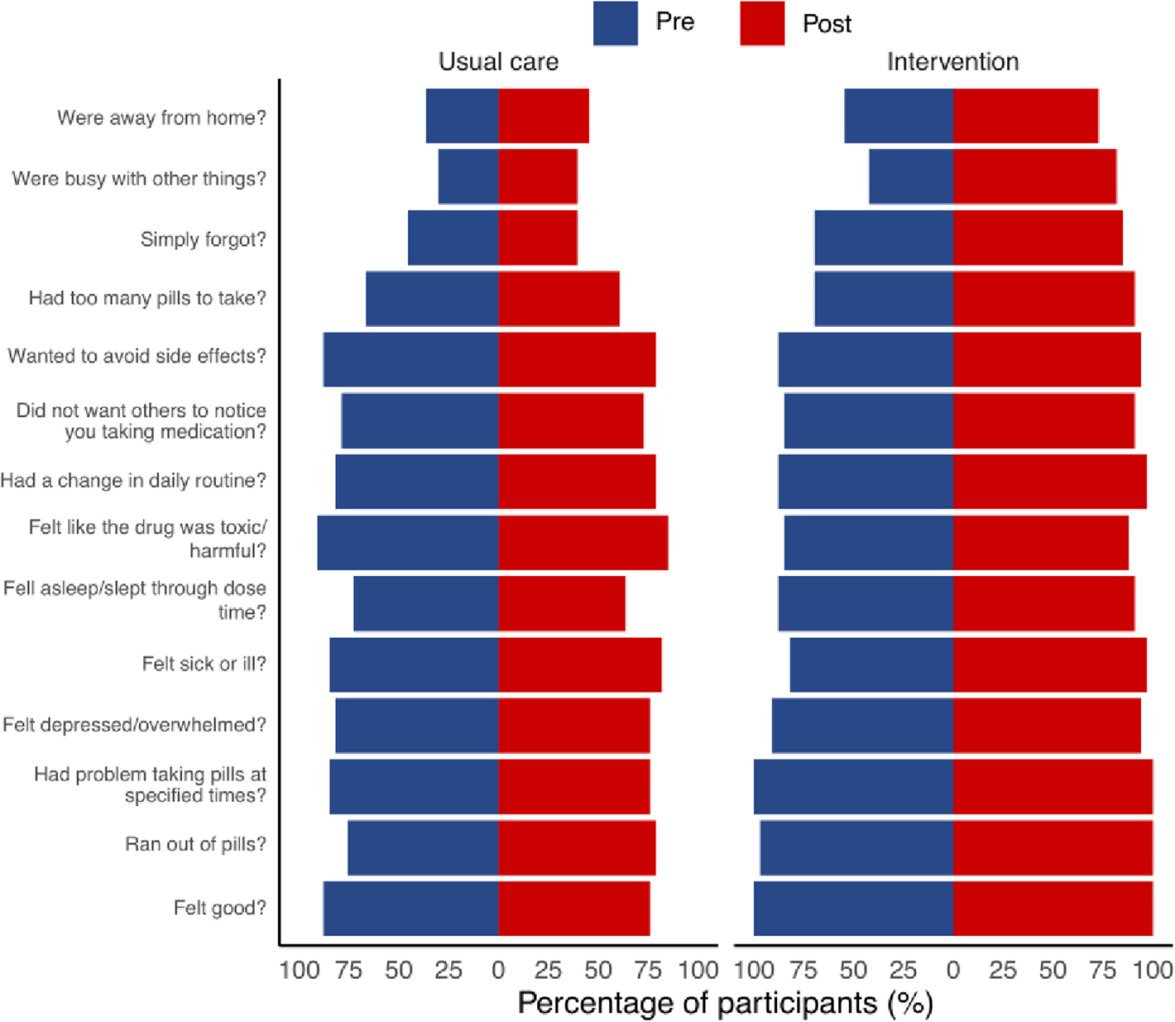
Factors related to adherence. Pre- and post-intervention plots illustrating the percentage of participants that reported reasons as ‘never’ causing them to miss their medications in the past month. An increased percentage post-intervention equates to fewer people missing their medication for this reason

The participants have rated 14 possible reasons for them having missed ART on the ‘often-sometimes-rarely-never’ scale where a higher response indicates that the reason is less likely to have contributed to missing the medications in the past month. Post-intervention, participants in the intervention group were 7.74 times more likely to report a higher response on the ‘often-sometimes-rarely-never’ scale (OR [95% CI] =7.74 [4.68–12.81], z = 7.96, *p* < 0.0001). Moreover, there was a statistically-significant association between higher responses and marital status (*χ*^2^ [2] =10.71, *p* = 0.0047), gender (*χ*^2^ [2] =16.83, *p* = 0.0002) as well as cause of infection (*χ*^2^ [2] =27.32, p < 0.0001) where unmarried participants were more likely to report higher responses (unmarried/married OR [95% CI] =2.88 [1.50–5.49]). Additionally, transgender participants were less likely to report higher responses (transgender/male OR [95% CI] =0.23 [0.11–0.48]). Moreover, participants who reported sexual transmission as the cause of infection were less likely to report higher responses compared to those who reported all other reasons for HIV transmission (other/sex OR [95% CI] =7.87 [3.19–19.42]). The supplementary file records further details regarding effect sizes. The most common reasons participants had ‘often’, ‘sometimes’, or ‘rarely’ missed their medications, were “being away from home”, “being busy”, “forgetful” and “having too many pills to take”. The reasons which had the response ‘never’ are presented in Fig. 4; these indicate the reasons which are not, or no longer, contributing to missed medications.

### Adherence self-efficacy and medication beliefs (section a, AACTG)

**Fig. 5:**
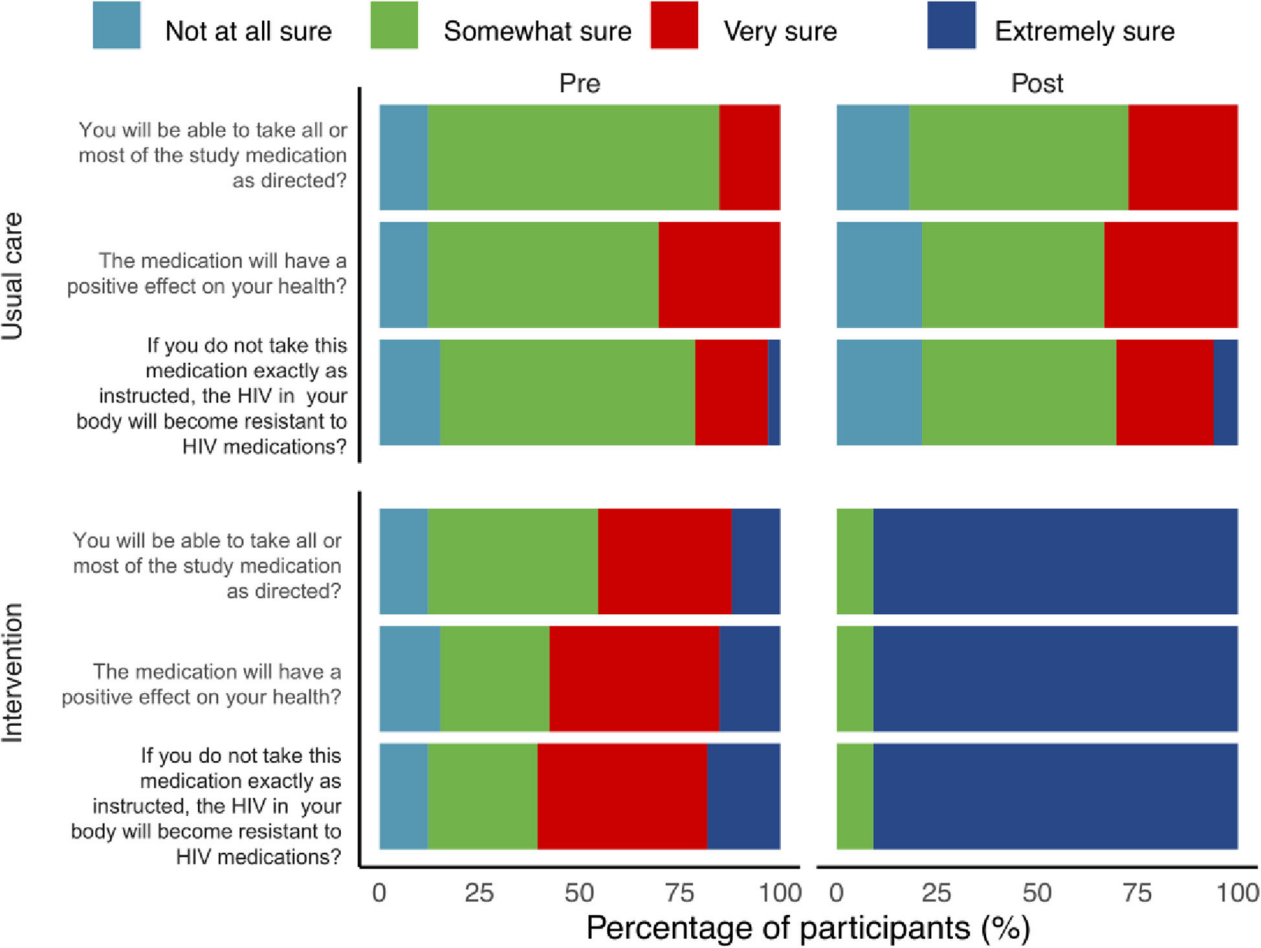
Results for adherence self-efficacy and medication beliefs. Pre- and post-intervention plots illustrating some changes

The responses to the three questions related to adherence self-efficacy and medication beliefs indicated that there were minimal changes in the usual care group (upper graphs) (Fig. 5). However, for the intervention group (lower graphs), adherence, self-efficacy and medication beliefs changed substantially where at post-intervention, a majority (> 75%) of the participants were ‘extremely sure’ that 1) they could take medication as directed 2) the medication would have a positive effect and 3) not taking their medicine would lead to ART resistance. The ordinal logistic regression model for this outcome measure failed to converge to a feasible solution as most of the participants in the post-intervention cell gave the same response as (‘never’) while leaving other cells empty.

## Discussion

To our knowledge, this RCT is the first to report on the effect of a pharmacist-led ART adherence intervention in Pakistan. The findings may be applicable to lower-middle income countries. Eight weeks following the delivery of a single 30-min session of pharmacist-led education and counselling, participants in the intervention group were statistically significantly more likely to report improved adherence. In addition, the intervention group had a statistically significant higher CD4 counts, indicating improved HIV disease status and immune system function [36]**.** Increased CD4 cell counts have been reported following other pharmacist-led adherence interventions [27, 45] Given the association between good ART adherence and optimal CD4 cell response [46], it is plausible that improved adherence levels contribute to improved CD4 counts and that the pharmacist-led intervention can ameliorate status of HIV. Although other clinical measures were not recorded, literatures would suggest that improved CD4 cell counts would make the individuals less susceptible to opportunistic infections [36] and have a lower risk of progression to AIDS and death [47].

The adherence findings were supported by non-systematic [23] and systematic [19, 48] literature reviews, which have shown the beneficial effects of education and counselling interventions on adherence to ART. Previous adherence interventions have been delivered by counsellors, nurses and community support workers and less commonly, by pharmacists [48]. Whilst the majority of research into pharmacist-led ART interventions has been observational or uncontrolled (pre-post) [32], two RCTs have previously been reported [29, 49], to which our results are comparable to. Ratbun et al. [29] conducted an RCT to explore the effect of a 12-week pharmacist-led intervention compared to the usual care. The intervention comprised of an initial 1.0 to 1.5-h session of education and counselling, a two-week follow-up visit and additional visits as well as telephone follow-up when required. The findings showed improved adherence and viral load post-intervention. In another study, Levy et al. [49] conducted a quasi-RCT and demonstrated improved self-reported adherence following a pharmacist-led intervention which comprised of a 2-h session of education and counselling, medication adherence aids and telephone advice as required.

Two further non-randomized controlled trials have investigated the effect of pharmacist-led counselling over five monthly-sessions and have shown 1) increased adherence [50] 2) a slower decline in adherence [51] and 3) decreased rates of hospitalization and opportunistic infections [50] in the intervention group when compared with the usual care. In comparison to our study, these pharmacist-led interventions were of longer duration and were provided at multiple time-points; thus, it is promising to see that the single 30-min intervention in our study significantly improved adherence and disease state indicating that 30-min is a minimum duration. An intervention which of short-duration is likely to be more cost-effective and therefore has better potential to be successfully implemented especially in lower- to middle-income countries like Pakistan. Having said this, a cost-benefit analysis was not conducted in our study and should be considered in future research.

The most common reasons for non-adherence were “being away from home”, “being busy” and “forgetful”, which is in line with literatures on ART [37, 52]. In the present study, the pharmacist tends to provide advice on the medications when travelling and when busy which are more likely to contribute to the fact that these factors have a low influence on non-adherence post-intervention (Fig. 3). The pharmacist-led intervention also improved 1) the participants’ beliefs in their ability to correctly take medications and 2) participants’ understanding on the benefits of ART and the risks of ART resistance. These changes may have improved motivation [53] and contributed to improvements in the overall adherence. Therefore, pharmacists and clinicians should ensure that these factors are addressed in any ART adherence program.

Socio-demographic factors are known to influence adherence. Economic costs of treatment were not a factor in this study, but travel costs may have limited the timely collection of ART medication. Transgender participants were more likely to report non-adherence, which is consistent with previous literature [54]. Detectable viral loads and failure to achieve viral suppression were associated with lower adherence among transgenders. Moreover, HIV related, and gender related stigma also plays important role in the adherence [54]. Those who reported sexual transmission as the cause to be less likely to be adherent to medication. Previous studies reported poor adherence is associated with unprotected sex. People who believe that they are less infectious when their blood viral load is undetectable, a result of treatment adherence, reduce condom use and increase unprotected sex. Contrary to other studies, although social support was associated with adherence, participants who had lower social support had higher adherence to medications post-intervention. Whilst this finding contrasts with literature in which higher social support has been associated with improved adherence [55], there are cases where non-disclosure of HIV status to family or friends led to some individuals having missed their medications due to fear or stigma of rejection [56, 57]. It is not known whether fear of stigma also contributed to the associations found in this study between marriages with lower adherence. Further qualitative research is needed to understand the underlying reasons for non-adherence specific to Pakistan to allow pharmacists to tailor their interventions more specifically to these needs.

Our study has some limitations, in which the study was performed under a limited budget in a resource-limited setting. CD4 counts were collected pragmatically from medical records and were therefore not available on some occasions. The lead investigator who delivered the intervention also administered the AACTG instrument and therefore was not blinded to group allocation. This may have produced some bias in measuring self-reported adherence and in the secondary measures. Fortunately, the possibility of bias is somewhat negated by the fact that the blinded CD4 cell count measures strongly support the data for adherence. Nevertheless, any future work, should ensure the AACTG instrument is administered by a blinded assessor.

It was noted that baseline levels of adherence differed between the usual care and intervention groups (45% vs 12% had missed medications in the previous 1–2 weeks, see Fig. 3), which was unexpected, given that participants were randomly allocated to groups. However, these baseline differences did not lead to biasness in the findings, as the statistical model corrected for baseline measures [58]. It has been suggested that permanent behavior change is likely to need long-term input [23] and therefore the eight-week follow up in this study was likely insufficient to determine lasting changes. Given ART medication needs to be taken long term, future researchers should monitor adherence over a longer period and consider options for providing low-cost follow up at regular intervals.

## Conclusions

The study demonstrated that a brief pharmacist-led education and counselling intervention can significantly improve ART adherence and HIV disease state in people with HIV living in Pakistan. In addition, pharmacist-led intervention also ameliorated participants’ beliefs in their ability to correctly take medications and improve their understanding on the benefits of ART as well as the risks of the development of ART resistance. This supports the integral role of pharmacists in patient care and the positive impact they can have on patient outcomes. Further research is needed to test the efficacy of intervention in other HIV centers and to explore ways to optimize its delivery.

## Supplementary Information

**Additional file 1.**

## Data Availability

All data generated or analyzed during this study are included in this current article. The datasets used and/or analyzed during the current study are available from the corresponding author on reasonable request.

## Abbreviations

AACTG: Adult AIDS Clinical Trials Group
ACTG: AIDS Clinical Trial Group
ART: Antiretroviral therapy
CD4: Cluster of differentiation 4
CONSORT: Consolidated standards of reporting trials
HIV: Human immunodeficiency
PIMS: Pakistan Institute of Medical Sciences
RCT: Randomized controlled trial
