# Supplementary material for: Pharmacist-led counselling intervention to improve antiretroviral drug adherence in Pakistan: a randomized controlled trial
Source: BMC Infect Dis. 2020 Nov 23;20:874. doi: 10.1186/s12879-020-05571-w (PMC7684945; doi:10.1186/s12879-020-05571-w)
Supplement: Supplementary file 1 — Additional file 1. [file 12879_2020_5571_MOESM1_ESM.pdf]

Statistical Analysis Report

Usman Rashid

05/09/2019

Data Summary

Variables included in the analysis

```
## 'data.frame':    132 obs. of  30 variables:
##  $ Pid           : Factor w/ 66 levels "1","2","3","4",..: 1 2 3 4 5 6 7 8 9 10 ...
##  $ Gender        : Factor w/ 3 levels "Male","Female",..: 3 1 1 1 2 2 1 1 1 1 ...
##  $ Age           : int  36 32 35 19 41 39 24 48 21 18 ...
##  $ Education     : Factor w/ 2 levels "Educated","Uneducated": 2 2 2 2 2 2 2 1 2 2 ...
##  $ Employment    : Factor w/ 2 levels "Employed","Unemployed": 1 2 1 1 2 2 1 1 1 2 ...
##  $ Marital_Status: Factor w/ 3 levels "Married","Unmarried",..: 2 2 1 1 1 1 2 2 2 2 ...
##  $ Cause         : Factor w/ 3 levels "Sex","Shared needles",..: 1 2 3 1 1 1 1 3 1 1 ...
##  $ Group         : Factor w/ 2 levels "Usual care","Intervention": 1 2 1 2 1 2 1 2 1 2 ...
##  $ Time          : Factor w/ 2 levels "Pre","Post": 1 1 1 1 1 1 1 1 1 1 ...
##  $ CD4_Count     : int  154 129 361 30 402 203 38 876 687 45 ...
##  $ A1            : Factor w/ 4 levels "Not at all sure",..: 2 2 3 2 2 4 1 1 2 3 ...
##  $ A2            : Factor w/ 4 levels "Not at all sure",..: 3 3 3 2 2 4 1 2 2 1 ...
##  $ A3            : Factor w/ 4 levels "Not at all sure",..: 2 3 3 2 2 4 1 2 2 1 ...
##  $ B1            : Factor w/ 4 levels "Very dissatisfied",..: 1 2 3 4 2 2 1 2 2 1 ...
##  $ B2            : Factor w/ 4 levels "Very dissatisfied",..: 1 4 3 4 2 4 1 2 2 2 ...
##  $ C1            : Factor w/ 4 levels "Never","Rarely",..: 2 3 2 1 1 1 1 1 1 1 ...
##  $ C2            : Factor w/ 4 levels "Never","Rarely",..: 2 1 2 2 1 1 4 2 1 1 ...
##  $ C3            : Factor w/ 4 levels "Never","Rarely",..: 3 1 1 1 1 1 4 1 1 1 ...
##  $ C4            : Factor w/ 4 levels "Never","Rarely",..: 1 1 1 1 1 1 4 3 1 3 ...
##  $ C5            : Factor w/ 4 levels "Never","Rarely",..: 1 1 1 1 1 1 1 1 1 1 ...
##  $ C6            : Factor w/ 4 levels "Never","Rarely",..: 3 1 1 1 1 1 4 1 1 4 ...
##  $ C7            : Factor w/ 4 levels "Never","Rarely",..: 2 1 1 1 1 1 4 1 1 1 ...
##  $ C8            : Factor w/ 4 levels "Never","Rarely",..: 1 1 1 1 1 1 1 1 1 1 ...
##  $ C9            : Factor w/ 4 levels "Never","Rarely",..: 2 1 3 2 1 1 4 4 1 1 ...
##  $ C10           : Factor w/ 4 levels "Never","Rarely",..: 1 1 1 1 1 1 4 1 1 1 ...
##  $ C11           : Factor w/ 4 levels "Never","Rarely",..: 2 1 4 2 1 1 4 1 1 3 ...
##  $ C12           : Factor w/ 4 levels "Never","Rarely",..: 1 1 2 1 1 1 4 1 1 1 ...
##  $ C13           : Factor w/ 4 levels "Never","Rarely",..: 1 1 3 1 1 1 4 1 1 1 ...
##  $ C14           : Factor w/ 4 levels "Never","Rarely",..: 1 1 1 1 1 1 1 1 1 1 ...
##  $ D             : Factor w/ 6 levels "Never","Past week",..: 5 6 3 6 1 1 2 5 1 4 ...
```

Sociodemographic Characteristics

Group	Characteristic	Levels	Mean	SD	N	Percentage
Usual care					33	
Intervention					33	
Usual care	Age		31.39	9.53		
Intervention	Age		36.18	12.24		
Usual care	Gender	Male			18	54.55
Usual care	Gender	Female			10	30.3
Usual care	Gender	Transgender			5	15.15
Intervention	Gender	Male			23	69.7
Intervention	Gender	Female			10	30.3
Usual care	Education	Educated			6	18.18
Usual care	Education	Uneducated			27	81.82
Intervention	Education	Educated			6	18.18
Intervention	Education	Uneducated			27	81.82
Usual care	Employment	Employed			15	45.45
Usual care	Employment	Unemployed			18	54.55
Intervention	Employment	Employed			10	30.3
Intervention	Employment	Unemployed			23	69.7
Usual care	Marital Status	Married			18	54.55
Usual care	Marital Status	Unmarried			14	42.42
Usual care	Marital Status	Divorced			1	3.03
Intervention	Marital Status	Married			21	63.64
Intervention	Marital Status	Unmarried			11	33.33
Intervention	Marital Status	Divorced			1	3.03
Usual care	Cause	Sex			23	69.7
Usual care	Cause	Shared needles			5	15.15
Usual care	Cause	Other			5	15.15
Intervention	Cause	Sex			25	75.76
Intervention	Cause	Shared needles			5	15.15
Intervention	Cause	Other			3	9.09
Usual care	B1	Very dissatisfied			7	21.21
Usual care	B1	Somewhat dissatisfied			10	30.3
Usual care	B1	Somewhat satisfied			13	39.39
Usual care	B1	Very satisfied			3	9.09
Intervention	B1	Very dissatisfied			5	15.15
Intervention	B1	Somewhat dissatisfied			8	24.24
Intervention	B1	Somewhat satisfied			14	42.42
Intervention	B1	Very satisfied			6	18.18
Usual care	B2	Very dissatisfied			9	27.27
Usual care	B2	Somewhat dissatisfied			8	24.24
Usual care	B2	Somewhat satisfied			7	21.21
Usual care	B2	Very satisfied			9	27.27
Intervention	B2	Very dissatisfied			4	12.12
Intervention	B2	Somewhat dissatisfied			9	27.27
Intervention	B2	Somewhat satisfied			10	30.3
Intervention	B2	Very satisfied			10	30.3

B1 and B2 correspond to the following questions from the AACTG instrument:

1. In general, how satisfied are you with the overall support you get from your friends and family members?
2. To what extent do your friends or family members help you remember to take your medication?

Primary Outcome 1 (CD4 Cell Count)

Data Visualisation

Following figure shows the box plots of the CD4 cell count for the two groups before and after the intervention.

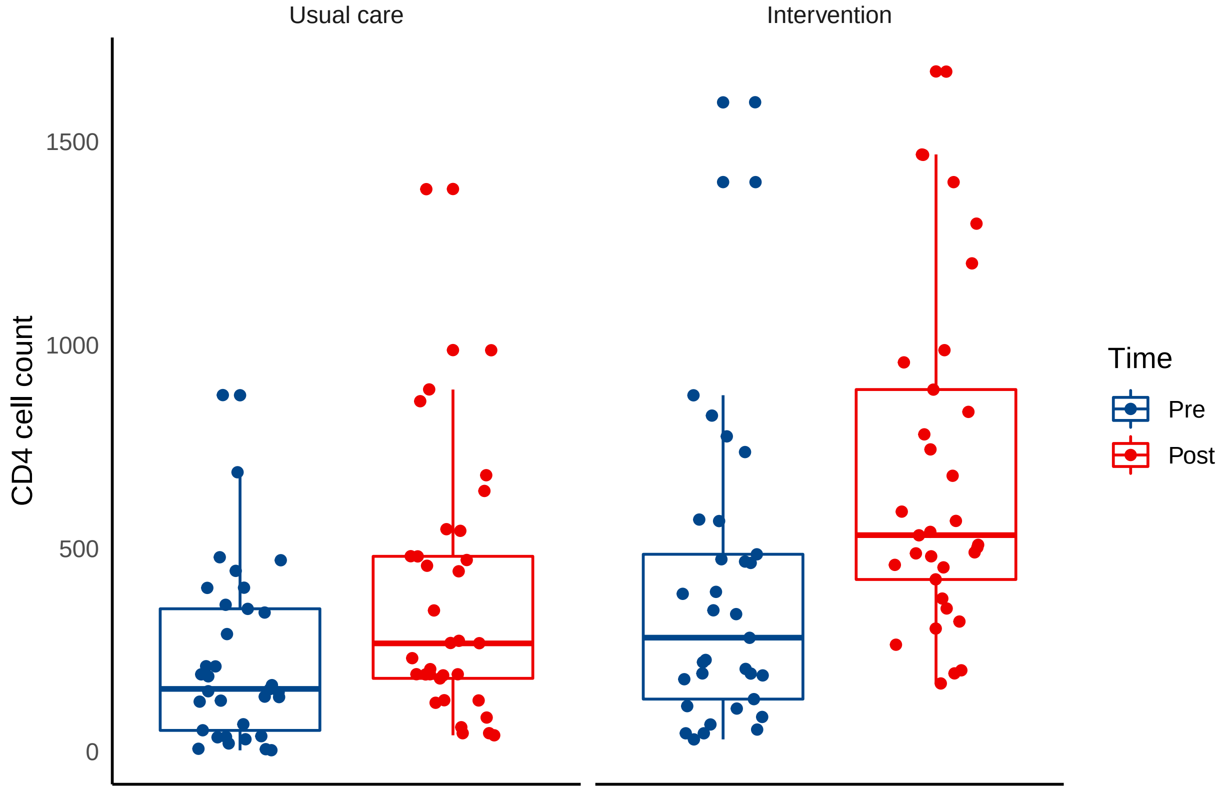

Means and Standard Deviations

Group	Time	Mean	SD
Usual care	Pre	221.7879	207.0415
Usual care	Post	370.3030	313.9302
Intervention	Pre	395.4848	372.2863
Intervention	Post	684.1818	411.8971

Statistical Model

```
lmModel <- lm(CD4_Count ~ CountPre + Group +
              B1 + B2 +
              Education + Employment + Marital_Status + Age + Gender + Cause,
              data = modelData)
```

Model Diagnostics

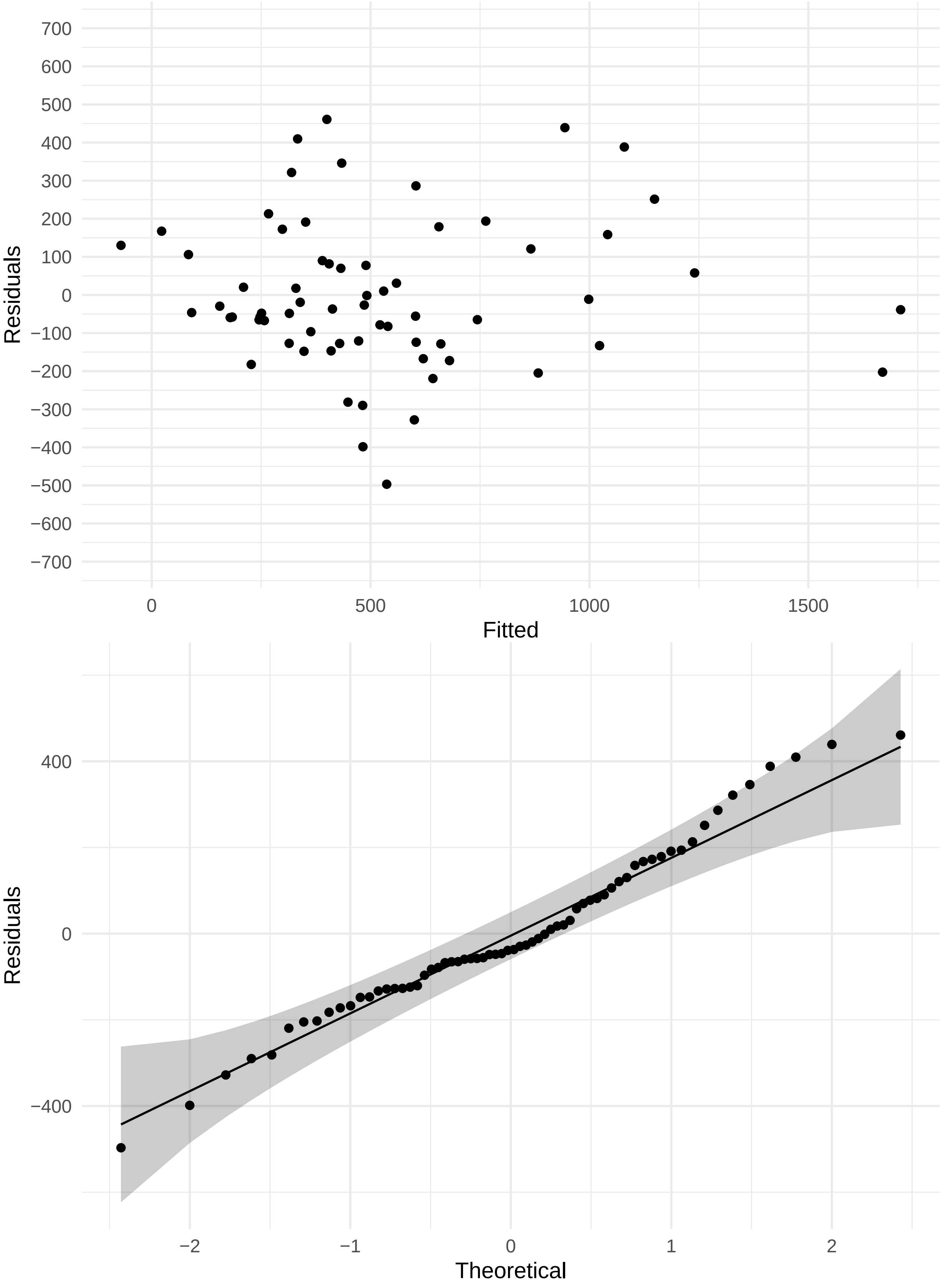

ANOVA Table and Group Differences

Variable	Sum Sq	Df	F value	Pr(>F)
CountPre	4182377.009	1	80.3311	0
Group	441517.280	1	8.4802	0.0054
B1	179067.009	3	1.1464	0.3399
B2	115008.692	3	0.7363	0.5355
Education	9602.158	1	0.1844	0.6695
Employment	5048.905	1	0.097	0.7568
Marital_Status	47795.184	2	0.459	0.6347
Age	102636.689	1	1.9713	0.1667
Gender	187908.397	2	1.8046	0.1755
Cause	15855.773	2	0.1523	0.8592
Residuals	2499084.393	48		

Contrast	Estimate	SE	DF	T-value	P-value	95% CI lower	95% CI upper
Intervention - Usual care	199.6693	68.5658	48	2.9121	0.0054	61.8086	337.53

Primary Outcome 2 (Self-reported Drug Adherence)

AACTG Section D: Time When Medication was Missed

Data Visualisation

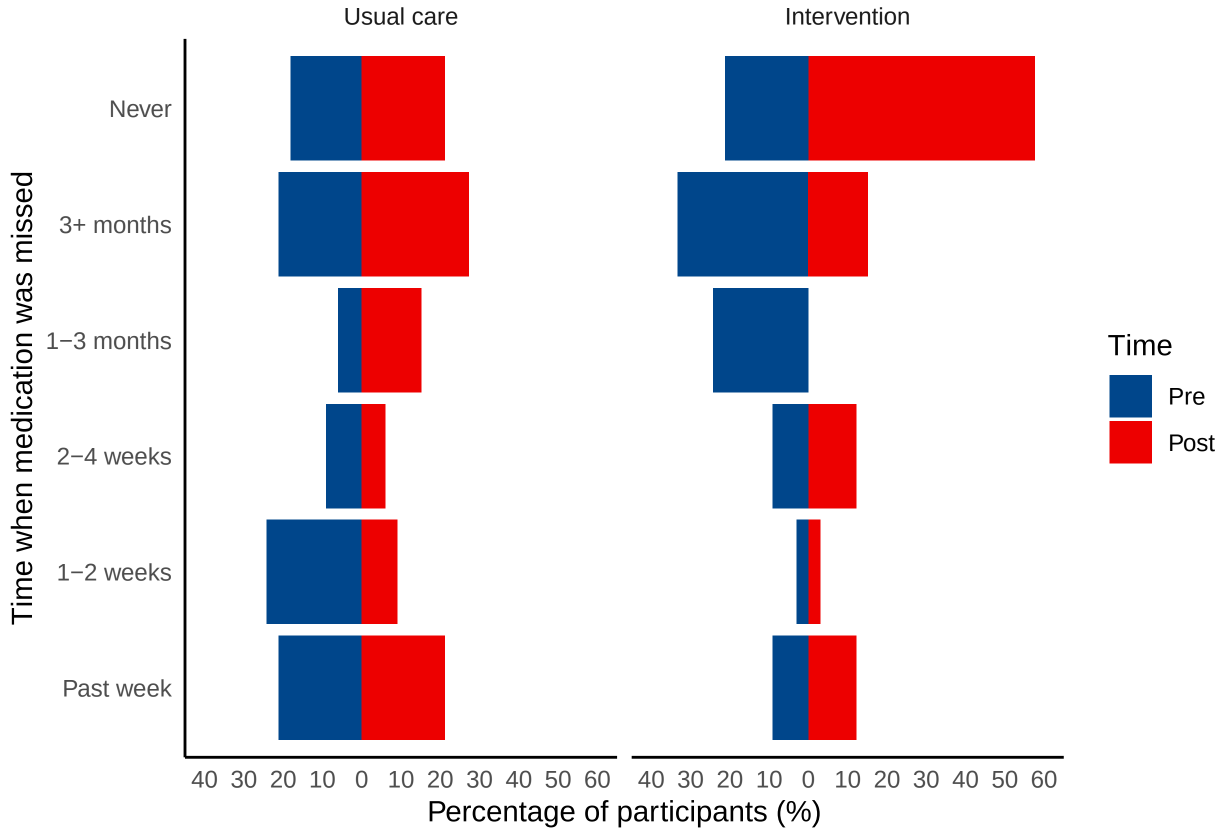

Statistical Model

```
modelData$Response    <- fct_relevel(modelData$Response,
                         c("Past week", "1-2 weeks", "2-4 weeks",
                           "1-3 months", "3+ months", "Never"))

modelData$ResponsePre <- fct_relevel(modelData$ResponsePre,
                         c("Past week", "1-2 weeks", "2-4 weeks",
                           "1-3 months", "3+ months", "Never"))

polrModel <- polr(Response ~ ResponsePre + Group +
                  B1 + B2 +
                  Education + Employment + Marital_Status + Age + Gender + Cause,
                  data = modelData,
                  Hess = TRUE,
                  method = "logistic")
```

ANOVA Table and Odds Ratio

Variable	LR Chisq	Df	Pr(>Chisq)
ResponsePre	21.9854	5	0.0005
Group	7.5834	1	0.0059
B1	4.9145	3	0.1782
B2	8.4443	3	0.0377
Education	2.2449	1	0.1341
Employment	1.2330	1	0.2668
Marital_Status	3.0879	2	0.2135
Age	0.1053	1	0.7455
Gender	3.3261	2	0.1896
Cause	9.1520	2	0.0103

Contrast	Odds Ratio	SE	Z-value	P-value	95% CI lower	95% CI upper
Intervention/Usual care	5.9569	0.6794	2.6268	0.0086	1.5730	22.5582
B2: Somewhat dissatisfied/Very dissatisfied	0.1830	1.3279	-1.2789	0.2009	0.0136	2.4704
B2: Somewhat satisfied/Very dissatisfied	0.0560	1.4973	-1.9245	0.0543	0.0030	1.0546
B2: Very satisfied/Very dissatisfied	0.8516	1.4340	-0.1120	0.9108	0.0512	14.1541
Shared needles/Sex	0.7230	0.9463	-0.3427	0.7318	0.1131	4.6200
Other/Sex	23.1431	1.1807	2.6609	0.0078	2.2878	234.1154

Note: Z and P values were obtained on the log odds ratio scale.

Secondary Outcomes (Self-reported Drug Adherence)

AACTG Section C: Reasons for Missed Medication

Data Visualisation

Percentage of participants across groups pre and post-intervention who said they never missed a medication for reasons C1 (Away from home) to C14 (Felt good).

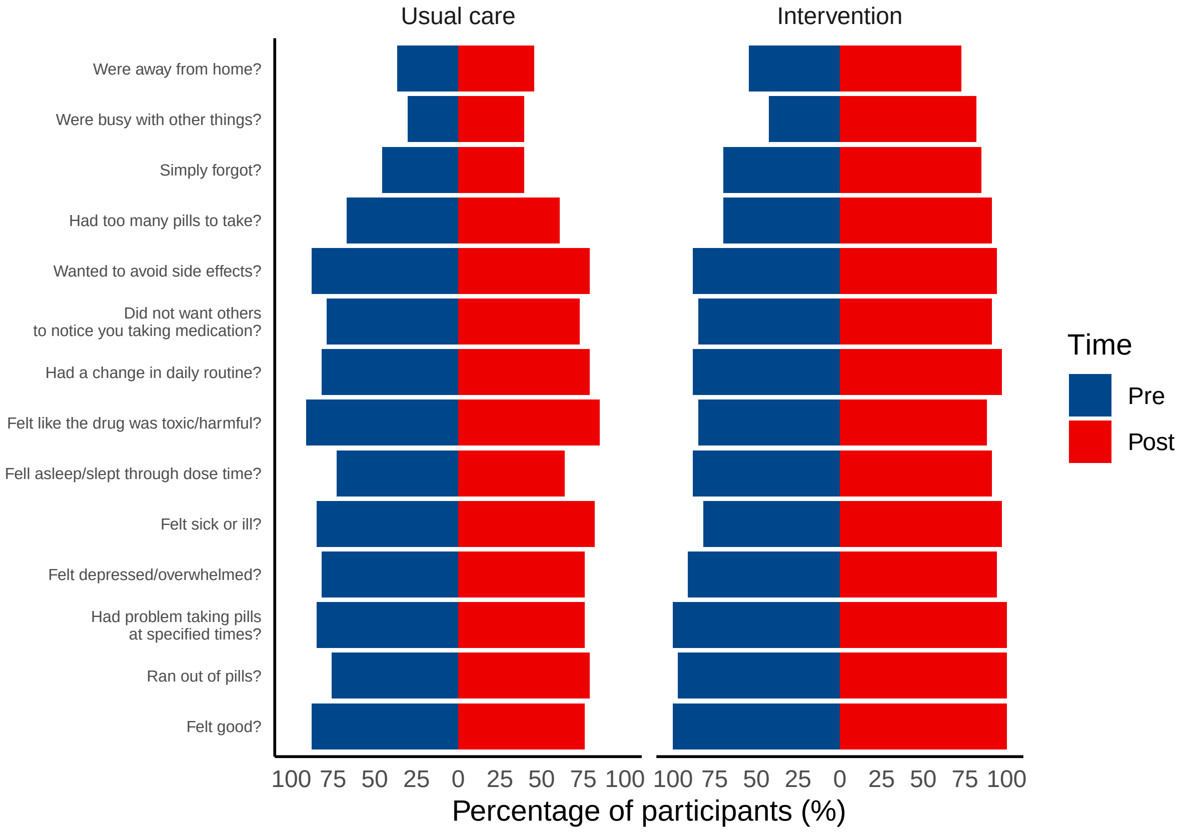

Statistical Model

```
modelData$Response    <- fct_relevel(modelData$Response,
                           c("Often", "Sometimes", "Rarely", "Never"))

modelData$ResponsePre <- fct_relevel(modelData$ResponsePre,
                           c("Often", "Sometimes", "Rarely", "Never"))

polrModel <- polr(Response ~ ResponsePre + Question + Group +
                  B1 + B2 +
                  Education + Employment + Marital_Status + Age + Gender + Cause,
                  data = modelData,
                  Hess = TRUE,
                  method = "logistic")
```

ANOVA Table and Odds Ratio

Variable	LR Chisq	Df	Pr(>Chisq)
ResponsePre	109.1533	3	0.0000
Question	30.7439	13	0.0037
Group	73.8569	1	0.0000
B1	3.5565	3	0.3135
B2	1.8192	3	0.6108
Education	1.0948	1	0.2954
Employment	2.6299	1	0.1049
Marital_Status	10.7104	2	0.0047
Age	0.0966	1	0.7560
Gender	16.8254	2	0.0002
Cause	27.3178	2	0.0000

Contrast	Odds Ratio	SE	Z-value	P-value	95% CI lower	95% CI upper
Intervention/Usual care	7.7414	0.2571	7.9595	0.0000	4.6769	12.8141
Unmarried/Married	2.8751	0.3304	3.1960	0.0014	1.5045	5.4945
Divorced/Married	0.8765	0.6334	-0.2081	0.8352	0.2533	3.0333
Female/Male	1.7393	0.3625	1.5267	0.1268	0.8546	3.5395
Transgender/Male	0.2257	0.3884	-3.8328	0.0001	0.1054	0.4832
Shared needles/Sex	1.8916	0.3528	1.8067	0.0708	0.9474	3.7769
Other/Sex	7.8715	0.4608	4.4779	0.0000	3.1905	19.4203

Note: Z and P values were obtained on the log odds ratio scale.

AACTG Section A: Adherence Self-efficacy and Medication Beliefs

How sure are you that:

A1: You will be able to take all or most of the study medication as directed?

A2: The medication will have a positive effect on your health?

A3: If you do not take this medication exactly as instructed, the HIV in your body will become resistant to HIV medications?

Data Visualisation

Percentage of participants across groups pre and post-intervention who gave a particular response to the above three questions.

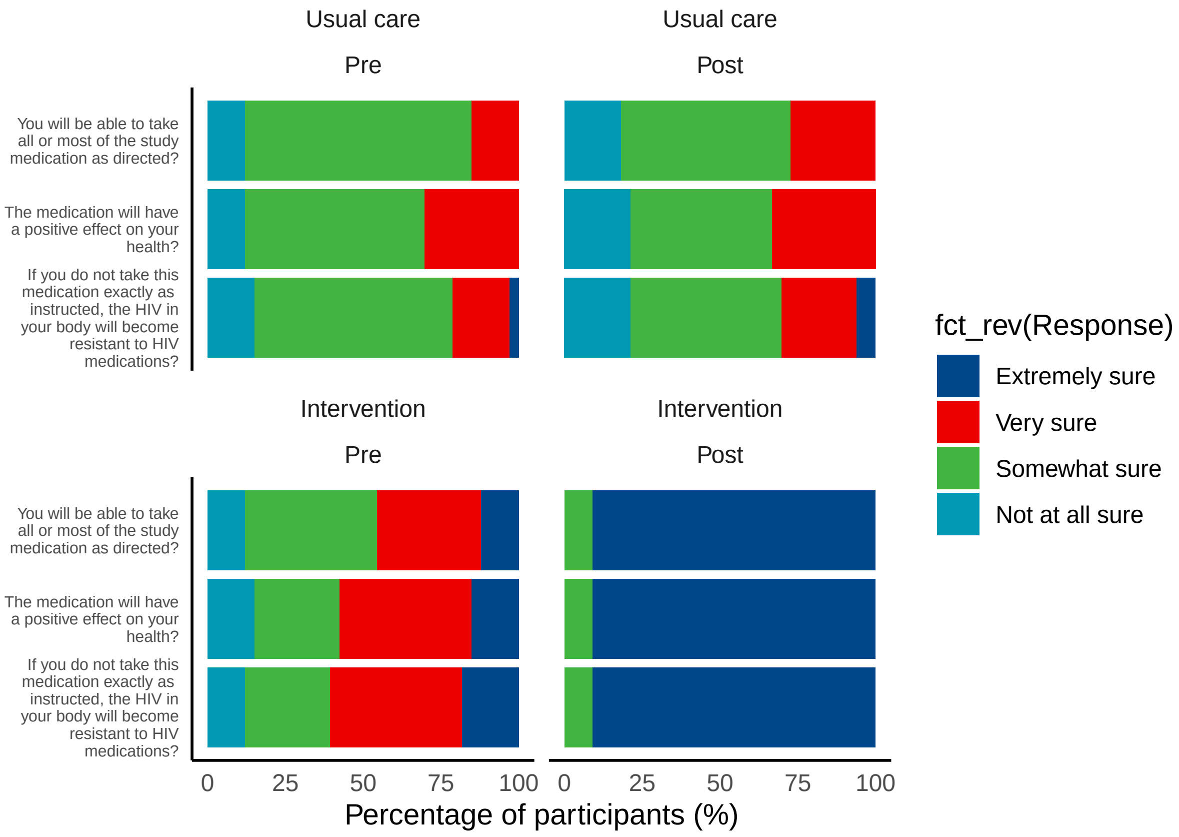

Statistical Model

We failed to setup a model to fit the data corresponding to adherence self-efficacy and medication beliefs as some of the categories were empty. The results are presented in the above figure overwhelmingly support the intervention group.
